# Disassociation of the SV40 Genome from Capsid Proteins Prior to Nuclear Entry

**DOI:** 10.1186/1743-422X-9-158

**Published:** 2012-08-10

**Authors:** Dmitry Kuksin, Leonard C Norkin

**Affiliations:** 1 Department of Microbiology, University of Massachusetts, Amherst, MA, 01003, USA; 2 Program in Molecular and Cellular Biology, University of Massachusetts, Amherst, MA, 01003, USA

**Keywords:** SV40, Polyomavirus, Nuclear entry

## Abstract

**Background:**

Previously, we demonstrated that input SV40 particles undergo a partial disassembly in the endoplasmic reticulum, which exposes internal capsid proteins VP2 and VP3 to immunostaining. Then, in the cytoplasm, disassembly progresses further to also make the genomic DNA accessible to immune detection, as well as to detection by an ethynyl-2-deoxyuridine (EdU)-based chemical reaction. The cytoplasmic partially disassembled SV40 particles retain some of the SV40 capsid proteins, VP1, VP2, and VP3, in addition to the viral genome.

**Findings:**

In the current study, we asked where in the cell the SV40 genome might disassociate from capsid components. We observed partially disassembled input SV40 particles around the nucleus and, beginning at 12 hours post-infection, 5-Bromo-2-deoxyuridine (BrdU)-labeled parental SV40 DNA in the nucleus, as detected using anti-BrdU antibodies. However, among the more than 1500 cells examined, we never detected input VP2/VP3 in the nucleus. Upon translocation of the BrdU-labeled SV40 genomes into nuclei, they were transcribed and, thus, are representative of productive infection.

**Conclusions:**

Our findings imply that the SV40 genome disassociates from the capsid proteins before or at the point of entry into the nucleus, and then enters the nucleus devoid of VP2/3.

## Background

Simian virus 40, as well as several other polyomaviruses (e.g. murine polyomavirus and BKV), are taken up into cells by virus-induced, caveola-mediated endocytosis [1-4]. The entry pathway of these viruses then follows a rather unusual route to the nucleus, by first passing through the endoplasmic reticulum (ER) [3,5].

Previously, we demonstrated that SV40 particles undergo partial disassembly in the ER, as shown by the finding that within that organelle the internal capsid proteins, VP2 and VP3, become accessible to immunostaining with antibodies [6]. More recently, we asked whether SV40 disassembly in the ER occurs to an extent that might also make the viral genome accessible to an antibody-based detection procedure. We found that the genomic DNA becomes accessible to each of two independent detection procedures, one based on detecting BrdU-labeled DNA with anti-BrdU antibodies and the other based on an EdU (ethynyl-2-deoxyuridine)-based chemical reaction, only after the partially disassembled SV40 particles emerge in the cytoplasm [7]. The cytoplasmic partially disassembled SV40 particles retain at least some of each of the three SV40 capsid proteins, as well as the viral genome. Thus, SV40 particles undergo discrete disassembly steps during entry that are separated temporally and topologically. First, a partial disassembly of the particles occurs in the ER, which causes internal capsid proteins VP2 and VP3 to become accessible to detection with antibodies. Then, in the cytoplasm, disassembly progresses further to also make the genomic DNA accessible to immune detection, as well as to an EdU-based procedure.

A key unanswered question regarding the unique SV40 entry pathway concerns the location where the viral genome might be completely released from the capsid components. Experimental results reported here imply that the SV40 genome disassociates from the internal capsid proteins VP2 and VP3 prior to, or at the point of nuclear entry, and then enters the nucleus without them.

## Results

We incorporated BrdU into parental SV40 genomes, and used anti-BrdU antibodies to detect exposure of the BrdU-labeled DNA within partially disassembled viral particles, as well as to detect released SV40 genomes, as previously described [7]. Exposed internal SV40 capsid proteins, VP2 and VP3, were detected using an antibody that recognizes a common epitope on these proteins [6]. Because we can not distinguish between VP2 and VP3 using this antibody, we use the designation “VP2/3” to refer to the two proteins it recognizes. Note that under our conditions of incorporating BrdU into viral genomes (i.e., at 1 μg/ml), there was no effect on the kinetics of SV40 entry [7]. Nor was there an effect on subsequent infectivity, as measured by expression of the SV40 large T antigen (LT, an early SV40 gene product) [7,8].

**Figure 1:**
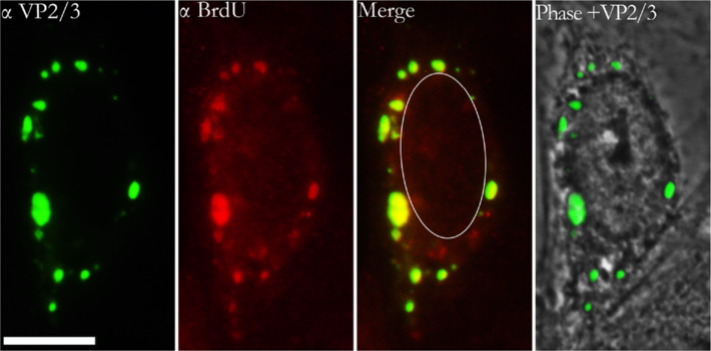
**A representative infected cell at 12 hours post-infection.** Perinuclear BrdU-labeled SV40 genomes are associated with VP2/3. BrdU-labeled SV40 genomes are seen in nuclei. However, parental VP2/3 is not seen in nuclei that contain viral genomes. The outline of the nucleus is delineated in the phase contrast image by the nuclear envelope and underlying nuclear lamina. It is further highlighted by the hashed white line drawn on the merge image. Samples stained for BrdU (Cy_3_) and VP2/3 (FITC). The size bar corresponds to 10 microns.

A representative 12-hour infection sample is shown in Figure 1. The outline of the nucleus is delineated in the phase contrast image by the nuclear envelope and underlying nuclear lamina. It is further highlighted by the hashed white line drawn on the merge image. Partially disassembled SV40 particles, which contain the exposed SV40 genome and exposed VP2/3, can be seen at perinuclear sites.

**Figure 2:**
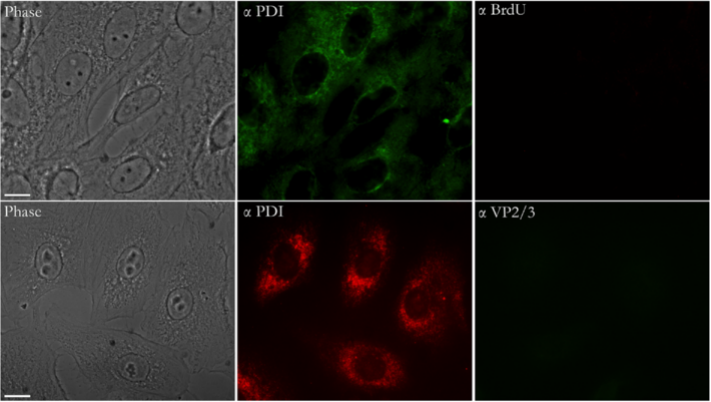
**BrdU stain is not seen in CV-1 cells mock-infected with control media.** Top panels: BrdU control. Control media was prepared by adding BrdU to regular media, which was then applied to non-infected cells. After 12 days (to mimic the virus labeling period) this media was harvested, spun down, dialyzed 3X, and then used to mock-infect control cells. These cells were fixed and immunostained at 12 hr for PDI (FITC) and BrdU (Cy_3_). Bottom panels: anti-VP2/3 control. As above, but the cells were fixed and immunostained at 12 hr for PDI (Cy_3_) and VP2/3 (FITC)The size bar corresponds to 10 microns.

**Figure 3:**
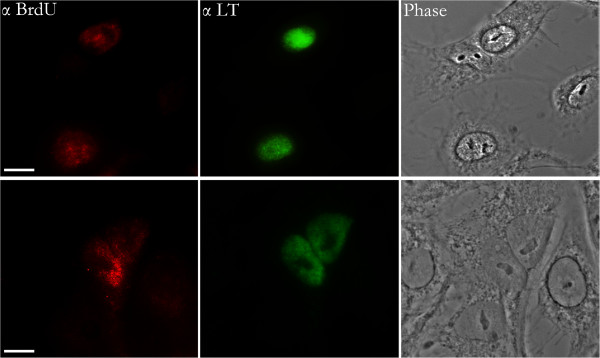
**LT-expression within nuclei containing BrdU-labeled SV40 genomes.** Two sets of representative cells, infected with BrdU-labeled SV40 and co-immunostained for BrdU (Cy_3_) and LT (FITC) at 12 hours post-infection. The size bar corresponds to 10 microns.

BrdU-labeled input SV40 DNA also can be seen in the nucleus of the cell in Figure 1. Although the immunostain of the nuclear BrdU-labeled DNA appears dispersed and relatively faint, as compared to the punctate BrdU immunostain seen in the cytoplasm, it is real. It was not seen in control cells, which were mock-infected with virus-free supernatant collected as a control during the BrdU-labeling of the virus (Figure 2). Moreover, a BrdU signal was not seen in infected cells prior to 12 hours post-infection (Figure 3), although VP2/3 is exposed at this time, in agreement with our earlier report [7]. The relative faintness of the nuclear BrdU stain (Figure 1) might be due to the physical nature of the genome as it enters the nucleus. For instance, the viral minichromosome, which is compact within the capsid, might be strung out as it enters the nucleus, in preparation for transcription and replication, thereby negatively affecting the concentration of the stain.

Importantly, no VP2/3 can be seen within the nuclei of SV40-infected cells at either 8 or 12 hours post infection (Figures 1, 3). What is more, the fact that the cytoplasmic VP2/3 stain was considerably more intense than the nuclear BrdU DNA stain supports the premise that VP2/3 of parental virus particles does not enter the nucleus with the viral genome but, instead, remains behind in the cytoplasm.

**Figure 4:**
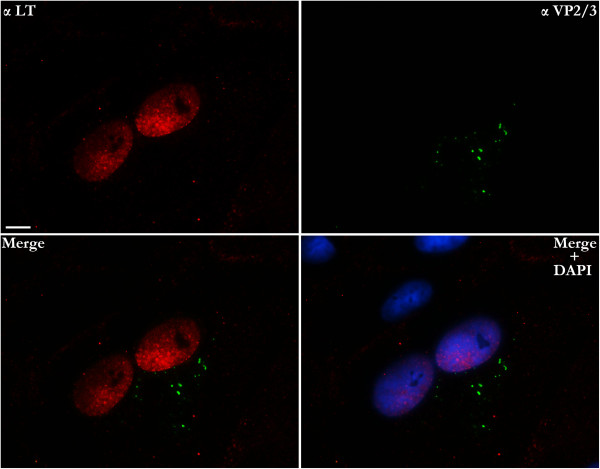
**Parental VP2/3 is not seen in nuclei of LT-expressing cells infected with BrdU-labeled SV40.** Cells infected with BrdU-labeled SV40 were fixed and co-immunostained for LT (Cy_3_) and VP2/3 (FITC) at 12 hours post-infection. Nuclei are labeled with DAPI in the bottom right panel. VP2/3-containing entities were readily seen in the cytoplasm and at the nuclear periphery of LT-expressing cells, but no VP2/3 was ever detected in the nuclei of these cells. The size bar corresponds to 10 microns.

In addition to the population of disassembly intermediates in which labeled viral DNA was found in association with VP2/3 (VP2/3^+^BrdU^+^), there also were cytoplasmic entities that stained positively for VP2/3, but which appeared to be devoid of DNA (VP2/3^+^BrdU^-^) (Figure 4, white arrows). Perhaps these VP2/3^+^BrdU^-^ entities are remnants of SV40 disassembly intermediates that released their genomes into the nucleus or, perhaps, into the cytoplasm (see Discussion). Consistent with the former possibility, BrdU-labeled parental SV40 DNA was seen in the nuclei of these cells, but not in other cells of the fields (Figure 4). Indeed, from three individual 12-hour sample sets, each infected with BrdU-labeled SV40, a total of 1500 cells were examined to ask whether the presence of cytoplasmic VP2/3^+^BrdU^-^ entities might correlate with nuclear BrdU immunostaining. Of 262 cells that contained one or more VP2/3^+^BrdU^+^ disassembly intermediates, we were able to ascertain that at least 210 of these cells also had at least one VP2/3^+^BrdU^-^ entity. Within that population of 210 cells, 131 cells (62%) were scored as staining positively for BrdU-labeled DNA in the nucleus. In contrast to the 12 hour samples, at 10 hpi we did not detect any VP2/3^+^BrdU^-^ entities, or nuclear staining for BrdU-labeled DNA (not shown).

Note that the intensity of the VP2/3 stain of the cytoplasmic VP2/3^+^BrdU^-^ entities is less than that of the VP2/3^+^BrdU^+^ entities. This might be due to some VP2/3 loss from these particles when they release their DNA for entry into the nucleus; a finding consistent with the likelihood that the viral DNA plays a role in maintaining the structural integrity of the disassembly intermediates [9].

**Figure 5:**
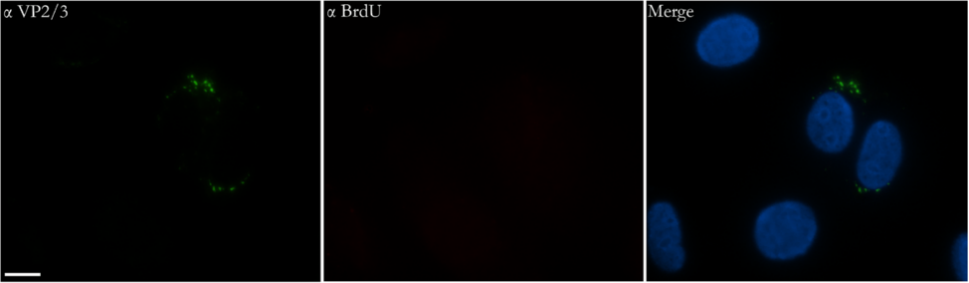
**BrdU immunostaining is not seen in CV-1 cells at 8 hours post infection, whereas cytoplasmic VP2/3 immunostaining is seen.** In the merge image, nuclei are highlighted with DAPI (blue). The size bar corresponds to 10 microns.

**Figure 6:**
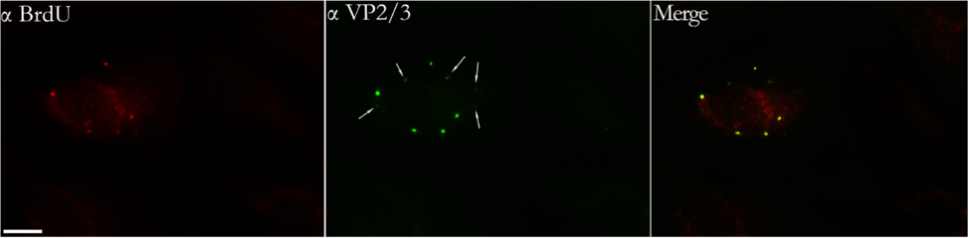
**BrdU-labeled SV40 DNA in the nuclei of cells containing VP2/3 **^**+**^**BrdU**^**- **^**viral entities.** Cells were infected with BrdU-labeled SV40, fixed at 12 hours post-infection, and immunostained for BrdU (Cy_3_) and VP2/3 (FITC). White arrows in the anti-VP2/3-stained image point to entities for which no BrdU is seen in the anti-BrdU-stained image. These are referred to as VP2/3^+^BrdU^-^ entities. The size bar corresponds to 10 microns.

To confirm that the nuclear BrdU-labeled SV40 genomes seen here are transcribed, and thus indicative of productive infection, we infected cells with BrdU-labeled SV40 and, at 12 hours post-infection, co-immunostained infected cell cultures for LT and for BrdU-labeled DNA. BrdU-labeled viral genomes indeed were detected in the nuclei of LT-producing cells (Figure 5). Additionally, in a separate experiment, cells infected with BrdU-labeled SV40 were co-immunostained for LT and VP2/3 at 12 hpi. VP2/3-containing entities were readily seen in the cytoplasm and at the nuclear periphery of LT-expressing cells, but no VP2/3 was ever detected in the nuclei of these cells at these early times (Figure 6).

## Discussion

It may be possible that a small number of VP2/3 molecules, which is below our level of detection, do accompany the viral genome into the nucleus. Nevertheless, we can not ignore the fact that among the more than 1500 cells examined, we never detected input VP2/VP3 in the nucleus. Thus, our experimental findings imply that the SV40 genome separates from VP2/3 preceding or at the point of nuclear entry and thus enter the nucleus devoid of these viral proteins.

There is substantial evidence that nuclear localization signals (NLSs) on VP2/3 might mediate the targeting SV40 disassembly intermediates to the nucleus, and perhaps mediate nuclear entry as well. For example, microinjecting anti-VP2/3 antibodies into the cytoplasm blocks SV40 infection, and SV40 virus-like particles (VLPs), which contain NLS-deficient VP3, enter cells normally, but do not transport their DNA into the nucleus [10,11]. In view of these findings, our initial working hypothesis was that exposed VP2/3 on the SV40 disassembly intermediates would target these particles to the nucleus, and also mediate the nuclear entry of the SV40 genomes. However, considering our current findings, we believe that while VP2/3 may target the SV40 disassembly intermediates to nuclear pore complexes (NPCs), SV40 capsid proteins do not mediate nuclear entry of the genome per se. The larger adenoviruses provide a precedent for such a state of affairs. Adenovirus, subviral capsids attach to the NPC via the hexon protein. Next, they recruit a cellular motor, kinesin-1, which facilitates both the uncoating of the viral genome and its entry into the nucleus, with capsid fragments being left behind in the cytoplasm [12].

Given our findings, we might ask the following. First, what factors might separate VP2/3 from the viral genome prior to nuclear entry? We can only speculate. Nevertheless, one possibility is that this stage of SV40 disassembly is facilitated by a cytoplasmic chaperone, such as cytoplasmic Hsp70. In this regard, Hsp70 binds to SV40 and disrupts murine polyomavirus in vitro [13,14]. Moreover, the premise, that a cytoplasmic chaperone might facilitate the separation of VP2/3 from the viral genome, may account for at least some of the BrdU immunostain seen outside the nucleus that does not colocalize with VP2/3. The relative diffuseness of this stain, in comparison to the particle-associated BrdU stain, is consistent with this notion. It is possible that some of these “naked” cytoplasmic viral genomes may be aborted nuclear entry events, rather than intermediates in the nuclear entry pathway.

Second, how might SV40 genomes be targeted to cross the nuclear membrane? The 5.2-kbp circular double-stranded SV40 DNA genome is associated with about 200 copies of cellular histones, all of which contain an NLS. Thus, it is tempting to hypothesize that these histones might mediate nuclear entry of the viral genome via the nuclear transport machinery. However, contrary to that premise, microinjected SV40 genomes, in complex with empty capsids, but not with histones, were reported to enter the nucleus (10).

## Conclusion

Results reported here imply that SV40 disassembly intermediates, which contain exposed internal capsid proteins VP2/3, as well as exposed SV40 genomes, arrive at the nuclear periphery, where the genome separates from the capsid components and enters the nucleus without them. Taken together with our earlier findings [6,7], input SV40 particles undergo discrete disassembly steps during entry that are separated temporally and topologically. First, a partial disassembly of the particles occurs in the ER, which causes internal capsid proteins VP2 and VP3 to become accessible to detection with antibodies. Then, in the cytoplasm, disassembly progresses further to also make the genomic DNA accessible to immune detection, as well as to an EdU-based chemical reaction. Finally, prior to or concomitant with nuclear entry, the viral genome disassociates from capsid components and enters the nucleus without them. Whereas NLSs on exposed VP2/3 may target the SV40 disassembly intermediates to the nuclear periphery, they do not appear to mediate nuclear entry of the viral genome. The means by which the SV40 genome is transported into the nucleus remains to be resolved.

## Methods

### Reagents

African Green Monkey kidney cells (CV-1) were purchased from ATCC. Dulbecco’s Modified Eagle’s Medium (DMEM), 5-Bromo-2-deoxyuridine (BrdU), Pen-Strep, and bovine serum albumin (BSA) were purchased from SIGMA-ALDRICH (Saint Louis, MO). Fetal Bovine Serum-premium select was from ATLANTA Biologicals (Lawrenceville, GA). Fluoromount-G was from Southern Biotech (Birmingham, AL.). Rabbit anti-VP2/3 was from A. Oppenheim (Jerusalem, Israel), mouse anti-BrdU was from Invitrogen (Carlsbad, California). Mouse and rabbit anti-SV40 large-T antigen were from Calbiochem - EMD4 Biosciences (San Diego, CA). (FITC)-conjugated donkey anti-rabbit IgG (H + L), and (Cy_3_)-conjugated goat anti-mouse IgG (H + L) were from Jackson ImmunoResearch Laboratories (West Grove, PA).

### Viral DNA labeling

Procedure for the incorporation of 5-Bromo-2-deoxyuridine (BrdU) into SV40 genomes was as previously described [7].

### Infections

150 μl of BrdU-labeled SV40 stock was absorbed onto CV-1 cells growing in 8-well Lab-Tek chamber slides (Nunc, Rochester, NY) for 1 h at 4°C. After one hour the cells were washed once with DMEM plus 10% FBS. Fresh warm DMEM plus 10% FBS was then added to the wells, and the chamber slides were incubated at 37°C and 5% CO_2_. Input multiplicity of infection in all experiments was approximately 1 PFU per cell. The infections were stopped by washing the chamber slides three times with 1 × PBS and fixed in 100% methanol at −20°C for 10 minutes.

Control media for all mock-infected controls was prepared to closely mimic infected-cell media as follows. BrdU was added to regular media, which was then applied to non-infected cells. After 10–12 days this media was harvested, dialyzed, and used to mock-infect control cells. Importantly, these mock-infected cells were grown concurrently with and processed identically to their infected counterparts, as previously described [7].

### Immunostaining

Fixed samples were washed three times with PBS, and once with 0.01% bovine serum albumin (BSA). All samples that were infected with BrdU-labeled SV40 were then treated with 20 KU (Kunitz units) of DNase (Sigma-Aldrich) per well for 20 min at 37°C. After 20 min, the cells were washed 3 times with 0.01% BSA solution. Samples were then incubated with primary antibodies for 1 h at 37°C and then washed three times with 0.01% BSA. The following primary antibodies were utilized in various combinations with each other: mouse anti-BrdU (1:100), rabbit anti-VP2/3 (1:1,000), mouse anti-SV40 LT (1:200), rabbit anti-SV40 LT (1:100). Following the incubation with primary antibodies, the cell were washed 3 times with 0.01% BSA solution and incubated with secondary antibodies for 50 min at room temperature. Secondary antibodies FITC-conjugated donkey anti-rabbit IgG (H + L) (1:100), and Cy_3_-conjugated goat anti-mouse IgG (H + L) (1:200) were used in conjunction with the appropriate primaries. After the final wash, the cells were mounted in fluoromount-G.

### Microscop

Immunofluorescence microscopy was carried out as previously described [7].

## Abbreviations

SV40: Simian virus 40; ER: Endoplasmic reticulum; EdU: Ethynyl-2-deoxyuridine; BrdU: 5-Bromo-2-deoxyuridine; LT: Large T antigen; NLS: Nuclear localization signal; NPC: Nuclear pore complex.

## Competing interests

The authors declare that they have no competing interests.

## Author’s contributions

DK carried out all experiments. LN conceived of the study. DK and LN participated in its design and coordination. LN prepared the first draft of the manuscript and both authors contributed to its final version.
